# Recreational Water and Infection: A Review of Recent Findings

**DOI:** 10.1007/s40572-014-0036-6

**Published:** 2015-01-22

**Authors:** Lorna Fewtrell, David Kay

**Affiliations:** Centre for Research into Environment and Health (CREH), Department of Geography and Earth Sciences, Aberystwyth University, Ceredigion, SY23 3DB UK

**Keywords:** Epidemiology, Quantitative microbial risk assessment, Exposure, Gastrointestinal illness, Water quality, Point and non-point source pollution

## Abstract

This paper reviews the latest evidence provided by epidemiological studies and quantitative microbial risk assessments (QMRAs) of infection risk from recreational water use. Studies for review were selected following a PubMed search for articles published between January 2010 and April 2014. Epidemiological studies show a generally elevated risk of gastrointestinal illness in bathers compared to non-bathers but often no clear association with water quality as measured by faecal indicator bacteria; this is especially true where study sites are impacted by non-point source pollution. Evidence from QMRAs support the lack of a consistent water quality association for non-point source-impacted beaches. It is suggested that source attribution, through quantified microbial source apportionment, linked with appropriate use of microbial source tracking methods should be employed as an integral part of future epidemiological surveys.

## Introduction

This paper reviews the recent literature related to recreational water use (in natural waters) and infection. It focuses on the results of both epidemiological studies and quantitative microbial risk assessments (QMRAs), published between 2010 and mid-2014 (i.e., covering the period since the last World Health Organization (WHO) examination of this issue [1]), although some supporting and earlier papers are cited to provide additional context. Although the focus here is on infection (excluding exposure to cyanobacterial toxins), it is important to balance these findings against the health benefits and increased well-being that can result from interacting with natural surface waters [2] and recreational water in particular.

Recreational water users can be exposed to a range of disease-causing microorganisms, including those naturally present in water. Many of the infectious agents of concern in recreational water, however, are the result of faecal pollution (especially human). Sources of faecal pollution include sewage, surface runoff, domestic animals, and wildlife. Surveillance data from the United States of America (USA) showed that, during 2009 and 2010, there were 24 recreational water disease outbreaks associated with the use of natural waters, although 11 of these were attributed to cyanobacterial toxins. Microbial agents implicated included *Campylobacter jejuni*, *E. coli* O157:H7, *Shigella sonnei*, *Cryptosporidium* spp., *Giardia intestinalis*, norovirus, and avian schistosomes, with the largest outbreak due to norovirus [3]. Outbreak data have also shown that attack rates can be high, with two United Kingdom (UK) outbreaks [4, 5] relating to swimming events reporting attack rates between 31 % (River Thames) and 85 % (Strathclyde Loch).

Recreational water epidemiology goes back over 60 years with early studies being conducted in both the USA [6] and the UK [7]. Early studies observed exposure-response associations between enterococci and *E. coli,* commonly used bacterial indicators of faecal contamination in water*,* and gastroenteritis among bathers. As a result, enterococci, *E. coli*, and other indicators have been widely used to monitor the quality of recreational waters. Since that time, more sophisticated studies have been conducted, exploring different indicators of faecal pollution, different water users, and different environmental waters. Studies reported in the period 1990-2010 have been used to develop guidelines [1, 8] and standards [9, 10] designed to limit the health risk from recreational water exposures. The studies employed two broad protocols, a prospective beach survey in which the exposure status of the respondents was self-determined and a randomised controlled trial in which exposure status was randomised. Water quality in these studies was quantified for a range of indicators using both culture and molecular methods, with the quality to which bathers were exposed being assigned by either aggregate water quality measures (e.g., a daily average) or by a series of participant-specific levels. The United States Environmental Protection Agency (USEPA) criteria are largely based on a prospective survey design and include molecular methods for bacterial enumeration, while the WHO guidelines and European Union (EU) standards are based on a randomised control trial approach with culture methods to define microbial exposure.

## Epidemiology

Papers published between 2010 and mid-2014, located through a PubMed keyword search, are summarised in Table 1. A variety of keywords were used, including recreational water, marine water, freshwater, health, gastrointestinal, infection, and water quality. Epidemiological studies focusing on infection in recreational water users (excluding swimming pool users) were selected for review. Epidemiological studies have revealed a number of health impacts associated with recreational water use, including gastrointestinal illness, respiratory infections, eye infection, ear, nose, and throat complaints, and skin problems. However, gastrointestinal illness is the most commonly identified problem and also has formed the rationale for water quality criteria world-wide, so it is emphasised in Table 1.

**Table 1:** Summary of recent recreational water epidemiological studies

Water type/Study name, first author	Design	Study location	Population	N (completing follow up)	Water quality	GI Results*		Comments
						Age group	Risk of GI illness (95 % CI)	
Freshwater								
CHEERS Dorevitch [11, 12•]	Prospective cohort	Chicago Area Waterways system (CAWS) & inland lakes and rivers in the Chicago area (‘General Use Water’ – GUW), USA. CAWS – extensive secondary effluent inputs. GUW – point and non-point source pollution	Participants (limited contact water recreators and non-water recreators) were recruited at the study sites	10,747 participants	Water quality results for the whole study period were not reported, but GM ENT and *E. coli* levels higher at CAWS (164 and 678 cfu/100 ml) compared to GUW sites (70 and 96 cfu/100 ml respectively)	All ages	Low contact recreators versus no water contact showed elevated AOR for both water types. CAWS: 1.46 (1.08-1.96) GUW: 1.50 (1.09-2.07). There was no significant difference between the two water types. Gastrointestinal illness was far more likely in those recreators who reported swallowing water: AOR 5.74 (2.05-16.04)	In the GUW, which were approved for full body contact, recreators reported head/face submersion more frequently than CAWS: speculation that the pathogen ingestion might actually be similar between the groups (i.e., at GUW, less pathogens per unit but more units ingested).
Marion [13]	Prospective cohort	East Fork Lake, Ohio, USA. Impacted largely by non-point source pollution	Recruitment at study beach	965 participants	Single daily water sample. Cultured *E. coli* Mean 95.1 cfu/100 ml	All ages	Bathers vs non-bathers: AOR 3.2 (1.1-9.0)	Exposure to water with *E. coli* concentrations greater than 11.3 cfu/100 ml presented a significantly elevated odds ratio for GI illness (AOR: 7.2, 95 % CI: 1.3-39).
Marine water								
Arnold [14]	Prospective cohort	Malibu, California, USA. Impacted by non-point source pollution	Recruitment at study beach	5,454 participants (2,559 bathers and 1,895 non-bathers) followed up at 10-19 days	5 sample points with samples taken twice daily. GM ENT: 3 cfu/100 ml; range 0.5 – 1,740 cfu/ml. GM *E. coli*: 13 cfu/100 ml; range 0.5-1,000	All ages	Results at 3 days Body immersion vs non-bathers: AOR 1.90 ( 1.17-3.06) Head immersion vs non-bathers: AOR 1.91 ( 1.17-3.14) Swallowed water vs non-bathers: AOR 2.86 ( 1.64-4.97)	Indicator bacteria were not consistently associated with swimmer illness. Used a variety of methods to enumerate ENT. Bather exposure was based on the average of the two daily samples taken from the sample point closest to the bather.
BEACHES Fleisher [15] Sinigalliano [16] Abdelzaher [17]	Randomised controlled trial	Miami, Florida, USA. Subtropical beach impacted by non-point source pollution	Participants were randomly assigned to either water or to beach-only exposure	1,303 adult participants, all of whom were regular bathers, followed up at 7 days.	Bather-specific water samples. Mean ENT: 71 cfu/100 ml	Adults	Head immersion vs non-bathers: OR 1.79 (0.94-3.43)	Individual samples were taken by bathers and used to determine their exposure. Evidence of a dose-response relationship was only found between skin illness and increasing ENT exposure.
Colford [18•]	Prospective cohort	Dana Point, California, USA. Beach impacted by urban runoff	Recruitment at study beach	9,525 participants followed up 10-14 days.	Samples collected 3 times daily from 5 sites. ENT measured using molecular and culture methods. ENT range < 2 - 41,000 cfu/100 ml	All ages	Risk of diarrhoea Body immersion vs non-bathers: AOR 1.38 (1.03-1.86) Head immersion vs non-bathers: AOR 1.46 (1.07-1.99) Swallowed water vs non-bathers AOR 1.90 (1.29-2.80)	The beach has a naturally occurring sand berm, which forms when there is low flow in the polluted stream draining into the ocean. Water quality in the bathing water, thus, varies markedly according to whether the berm is open or closed. There was some association seen between cultured ENT and one of the qPCR methods of ENT analysis and GI illness among swimmers swallowing water on ‘berm-open’ days.
Cordero [19]	Prospective cohort	Luquillo, Puerto Rico. Tropical beach impacted by potential point and non-point source pollution	Recruitment at study beach	1,457 participants followed up at 10-12 days.	3 samples daily from 6 sites. GM ENT cfu/100 ml Summer: 1.5 Autumn: 2.1	All ages	Bathers vs non-bathers: AOR 0.88 (0.47-1.63)	Very clean water.
Greek bathers cohort study Papastergiou [20, 21]	Prospective cohort	3 beaches (A, B & C) in Greece with ‘excellent’ water quality, although there were some known non-point source sewage inputs	Greek beach-goers attending study beaches. Non-bathers (non-beach-goers) were recruited through a random phone survey	3,796 bathers and 571 non-bathers	1 sample daily from 7 sites. GM (range) cfu/100 ml ENT: A: 5.6 (0-1380) B: 2.8 (0-74) C: 2.5 (0-15)	All ages	Bathers vs non-bathers: OR 3.60 (1.28-10.13)	Bathers were also significantly more likely to report respiratory, eye, and ear infections. Illness was not associated with the measured faecal indicator bacteria.
Harder-Lauridsen [22]	Retrospective cohort	Amager Beach Park lagoon, Copenhagen, Denmark	Recruited by post-event questionnaire sent to triathlon participants	1,769 participants. 838 in 2010 followed up at 4 weeks. 931 in 2011 followed up at 2 weeks	Modelled water quality. 2010 ENT peak 6 × 10^3^ cfu/100 ml *E. coli* range 1.4 × 10^4^ – 2.6 × 10^4^ cfu/100 ml 2011 ENT < 200 cfu/100 ml *E. coli* < 500 cfu/100 ml	Adults	2010 participants vs 2011 participants: RR 5.0 (4.0-6.39). 2010 participants swallowing water vs 2010 not swallowing water: RR 2.5 (1.8-3.4)	Logistic regression was used to estimate the relationship between illness and water quality. The authors report that self-reported illness in the different swim groups followed the concentration of *E. coli* to which the swimmers were exposed.
NEEAR Wade [23]	Prospective cohort	3 beaches, Mississippi, Rhode Island & Alabama, USA known to be impacted by treated sewage	Recruitment at study beaches	6,331 participants; 2,644 bathers and 3,687 non-bathers followed up at 10 to 12 days.	3 samples daily from 6 sites. ENT GM (range) cfu/100 ml: Edgewater 7.2 (0.1-920) Fairhope 21 (0.1-3000) Goddard 3.6 (0.1-960)	All ages	Risk of GI illness was expressed relative to a log_10_ increase in indicator density ENT cfu: AOR 1.16 (0.84-1.61) ENT PCR: AOR 2.56 (1.29-5.11)	There was a relationship seen between the daily average level of ENT (measured using PCR) and also *Bacteroides* and the risk of GI illness.
Sánchez-Nazario [24]	Prospective cohort – pilot study	3 tropical beaches, Puerto Rico of Blue Flag standard with known point and non-point source faecal pollution	Recruitment at study beaches	641 participants; 552 bathers and 89 non-bathers followed up at 8-10 days	8 samples daily (taken at 3 hour intervals over a 24 hour period) GM (range) cfu/100 ml ENT: 39 (1-378) *E. coli*: 12 (1-200)	All ages	Bathers vs non-bathers: AOR 1.61 (0.73-3.55)	There were significant differences between bathers and non-bathers for overall respiratory symptoms - AOR 1.43 and skin symptoms – AOR 1.75.
Yau [25]	Prospective cohort	Avalon Beach, California, USA, polluted	Recruitment at study beach	6,165 participants, followed up at 10-14 days	3 samples daily from 4 locations. ENT: <2 - >10,000 cfu/100 ml	All ages	Bathers (any contact) vs non-bathers AOR 1.27 (0.88-1.82). Swallowed water vs non-bathers AOR 1.51 (0.93-2.45)	Relationship between enterococci and bathers swallowing water seen when allowing for a submerged groundwater discharge.

* Exact definition of gastrointestinal (GI) illness varies by study GM – geometric mean ENT – enterococci AOR – Adjusted odds ratio RR – Relative risk cfu – colony forming units

qPCR – quantitative Polymerase Chain Reaction

The studies varied in how they defined bathers, with some gathering information on different degrees of exposure (splashed, body immersion, head immersion, swallowed water) and duration of recreational activity. The definition of gastrointestinal illness also varied between studies, with some being more rigorous than others. The non-exposed or control group was usually recruited at the same location, with the main exception being the study by Papastergiou [21] where non-bathers were recruited by telephone survey. Most recreators and non-recreators were self-selecting. The BEACHES study [15–17], however, randomised participants into bathing and non-bathing groups.

The ways in which water quality was assessed also varied between studies. A range of different possible indicators of faecal contamination were assessed (although all of the studies, except for Marion et al. 2010 [13], included enterococci measurements), using different methods of analysis (e.g., culture, plate counts and/or most probable number, and molecular) with different bather attribution techniques (e.g., personal sampling, daily averages, single daily sample).

While a statistically significant increase in gastrointestinal illness was not seen in all of the epidemiological studies, only Cordero et al. 2012 [19] reported a lower likelihood of gastrointestinal illness in bathers compared to non-bathers (AOR 0.88). Studies targeting beaches polluted by point sources of faecal pollution (e.g., Wade et al. 2010 [23]) generally reported results supporting earlier work (i.e., a relationship between gastrointestinal illness and microbial indicator concentration).

The importance of being aware and accounting for local conditions is illustrated by two studies [18•, 25] where the risk of swimming-related gastrointestinal illness was elevated when stream [18•] and submarine groundwater discharge [25] was high. In both cases there was a relationship between water quality and gastrointestinal illness only when the pollution sources were discharging to the recreational water.

### Non-point Source Pollution

Until relatively recently, epidemiological studies have tended to focus on bathing locations with known point sources of human faecal pollution and it is these studies that established relationships between faecal indicator bacteria and the risk of gastrointestinal illness [26, 27].

Non-point sources can include storm water runoff, septic tank drainage, sand re-suspension, animal faecal inputs, and human bather shedding and, thus, may or may not contain human faecal inputs.

Of the ten studies summarised in Table 1, five focus on non-point source-impacted locations [13–17, 18•, 20, 21]. The only study to address freshwater non-point sources [13] identified a relationship between gastrointestinal illness and *E. coli* for concentrations above 11 cfu/100 ml. In the marine studies, generally, where there was a significant difference in gastrointestinal illness between bathers and non-bathers [14, 18•, 21], no relationships were seen between the risk of illness and water quality parameters (this was also true of an earlier study [28]). The one exception is the study by [18•], where there was a suggestion of a relationship between enterococci (measured by culture or molecular methods) and the risk of diarrhoea but only on ‘berm-open’ days, when polluted stream water was discharged to the marine bathing area.

### Bathers as a Source of Non-point Pollution

It has been suggested that bathers may act as a source of pollution in their own right, either by shedding microorganisms from their bodies or by stirring up polluted sand and sediment.

Papastergiou [20, 21] did not find a relationship between gastrointestinal illness and indicator bacteria (Table 1) at three beaches with ‘excellent’ water, but they did find an association between bather density and both gastrointestinal [OR 2.13, 95 % confidence interval (CI) 1.95-3.79] and respiratory illness (OR 2.99, 95 % CI 1.64-5.46). Beach A (with a median bather density of 1.41 bathers/100 m^3^) was used as the baseline and compared to illnesses reported by bathers at beaches B & C (median densities of 2.91 and 21.65 bathers/100 m^3^, respectively). It was presumed that the increased illness was caused by bather to bather transmission via the water (gastrointestinal illness) and air (respiratory pathogens).

Graczyk [29] found a significant relationship between bather numbers at a marine beach in the USA and turbidity. The detection of three selected pathogens was significantly correlated with both enterococci levels and bather density. It was thought that the bathers were causing re-suspension of bottom sediments, which resulted in the elevated levels of enterococci and pathogens seen when bather numbers were high.

### Sand

Sand has been suggested as a possible sink for faecal pollution and, in a recent review [30], it was noted that faecal indicator bacteria were usually found in significantly higher concentrations in sand than in water samples. Sand could, therefore, present an infection risk in its own right [31] as well as potentially negatively impacting water quality. One study in Puerto Rico (Sánchez-Nazario et al. 2014 [24]) took sand samples and reported geometric mean enterococci levels of 97 cfu/100 g and 39 cfu/100 ml in sand and water, respectively. The ‘BEACHES’ study in Florida also collected and analysed sand samples prior to the epidemiological study, with enterococci levels of 4 – 1,088 cfu/g reported, along with the isolation of *Cryptosporidium* spp. [32]. As part of The National Epidemiological and Environmental Assessment of Recreational Water Study (NEEAR study), wet sand samples were taken from two of the beaches (Fairhope and Goddard) and participants were asked about sand contact and water-related activities [33•]. Digging in the sand was strongly associated with swimming (81 % of swimmers reported digging in the sand compared to 19 % of the non-bathers). However, after adjusting for swimming, sand enterococci concentration determined by culture methods was positively associated with both sand digging and gastrointestinal illness (AOR 1.7; 95 % CI 1.1-2.7).

### Animal Inputs

Where there are non-point source inputs, it is difficult to establish whether pollution is due to animal or human sources and it is known that animal inputs can contribute substantially to faecal indicator loads. Converse [34], for example, found that gull removal (using dog harassment techniques) led to notable improvements in beach water quality, with a 50 % reduction in gull numbers associated with a 38 % decrease in enterococci concentration. Wang [35], on the other hand, examined a non-point source-impacted beach in Miami-Dade County, USA and estimated the contributions to enterococci load made as a result of bird and dog visitations (the only beach in the county allowing dogs). In this case, gulls were found to contribute relatively little to the load (with estimates varying between 10^3^-10^5^ cfu/day), while dogs were responsible for a much greater estimated load (10^9^-10^10^ cfu/day). Dufour [36] reviewed a number of epidemiological studies, all conducted before 2010, and found that there was no evidence for associations between swimming-associated gastrointestinal illness and exposure to natural recreational water polluted with faeces from non-human sources.

### Use of Molecular Methods for Indicator Enumeration

Culture techniques for the enumeration of indicator bacteria tend to take a long time to produce results, with an incubation period of at least 24 hours, and so a number of more rapid techniques are being investigated in the hope that data can be produced more quickly in order to improve management decisions. It is, thus, becoming fairly common for molecular (especially quantitative polymerase chain reaction – qPCR) as well as traditional culture methods to be used for indicator analyses in epidemiological studies [14–17, 18•, 19, 23, 28]. Studies typically report quite different results depending on the method of analysis [37] and, where results are given for enterococci for both culture and PCR methods, greater concentrations are reported with PCR.

Some epidemiological studies, principally at point source-polluted locations, have reported an association between enterococci measured using PCR and gastrointestinal illness [18•, 23, 38, 39]. The clearest relationships are those reported by Wade [23, 38, 39] who considered a number of beaches impacted by human sewage. At four freshwater beaches [38, 39], an association was found between the daily average log_10_ qPCR cell equivalents for enterococci and gastrointestinal illness in water users (AOR 1.26; 95 % CI 1.06-1.51), with a stronger association seen for children aged ten and younger (AOR 1.69; 95 % CI 1.24-2.30). In the study of three marine beaches [23] (Table 1), a log_10_ increase in daily average enterococci measured by PCR was found to result in an increased AOR of 2.56 (95 % CI 1.29-5.11). Enterococci measured by culture were also positively associated with gastrointestinal illness, but the association was not statistically significant. Unlike the freshwater sites, children at the marine beaches did not show increased susceptibility to illness with exposure to enterococci. The water quality and gastrointestinal illness association seemed to strengthen when swallowing water (AOR 8.9; 95 % CI 2.2-37) or spending over 90 minutes in the water (AOR 6.4; 95 % CI 1.2-33).

## Quantitative Microbial Risk Assessment (QMRA)

This is a fairly new tool for those interested in the likely health impacts related to recreational water use and it allows for exploration of different scenarios; it can be used to augment and complement recreational water epidemiological studies.

Table 2 summarises recent recreational water QMRAs by the four QMRA steps, as follows: hazard identification, exposure assessment, dose-response assessment, and risk characterization. Studies were identified through a PubMed search using keywords similar to those for the epidemiology search (with the addition of microbial, exposure, and risk assessment) and selected to illustrate a number of relevant questions. As can be seen, recent examples employ a range of different approaches and explore a variety of areas, including those that would be difficult to subject to an epidemiological study. They do, however, require a number of assumptions and they are limited by the pathogens for which dose-response relationships are available.

**Table 2:** Summary of recent recreational water QMRAs

Question/Hazard	Exposure	Dose-response	Risk characterization	First author & Ref
What sources of faecal contamination are likely to represent the greatest risk of infection?				
Pathogens associated with seagull faeces Pathogens associated with primary treated effluent	Ingestion of 35 cfu/100 ml enterococci derived from either seagull or human sources.	*Campylobacter jejuni*, *Salmonella enterica*, *Cryptosporidium parvum*, *Giardia intestinalis*, Norovirus (Norwalk virus)	Probability of GI illness after a single exposure to recreational water contaminated by: Seagulls 3.5 × 10^-5^ Sewage effluent 3.1 × 10^-3^	Schoen [40]
Pathogens derived from a range of non-human (gulls, pigs, chickens, cattle) and human sources (raw and secondary treated sewage)	Ingestion of 35 cfu/100 ml enterococci and 126 cfu/100 ml *E. coli* derived from human and non-human sources.	*Campylobacter jejuni*, *E. coli* O157:H7 *Salmonella enterica*, *Cryptosporidium* spp., *Giardia lamblia*, Norovirus (Norwalk virus)	Probability of GI illness from ingestion of water containing fresh faecal pollution at defined faecal indicator bacteria densities. Cattle and human sources gave similar expected levels of illness, with mean estimates generally above the benchmark illness level of 0.03 probability of illness. Risks from the other faecal sources were much lower.	Soller [41]
What pathogens are likely to cause the illness rates seen in a freshwater epidemiological study?				
Levels of illness or pathogen concentrations were ascribed using two different approaches: Health-based approach, where it was assumed that swimming-associated illnesses occurred in the same proportion as known illnesses in the USA due to all non-foodborne sources; Effluent-based approach, where pathogens were assumed to occur in recreational waters in the same proportion as in disinfected secondary effluent.	Swimmers were exposed to levels of faecal indicator bacteria seen in the epidemiological study [38, 39]. Freshwater sites (Great Lakes). Volume of water ingested was based on a point estimate of 33 ml [42].	*Campylobacter jejuni*, *E. coli* O157:H7 *Salmonella enterica*, *Cryptosporidium* spp., *Giardia lamblia*, Adenovirus, Norovirus, Rotavirus	Human enteric viruses, and norovirus in particular, could have accounted for the vast majority of the observed swimming-associated GI illness seen in the epidemiological study.	Soller [43]
What is the risk of GI illness from tropical marine waters impacted by stream discharge?				
Pathogens measured in stream discharge	Volume of water ingested [42]; natural log normal distribution mean 2.92, standard deviation 1.43. Exposure at tropical beaches impacted by stream discharge	*Campylobacter jejuni*, Non-typhi *Salmonella,* Adenovirus 4, Echovirus 12, Norwalk virus	The median risk of GI illness risk ranged between 0 and 21/1,000 depending upon the location. The illness risks from exposure to viruses were generally orders of magnitude greater than the risks from bacterial exposure	Viau [44]
What is the risk of illness from specific pathogens?				
Measured concentrations of *Cryptosporidium* and *Giardia* in lakes in The Netherlands.	Volume of ingestion, frequency (average 7 to 8 times a year) and duration of swimming (average 54 to 79 minutes) to freshwater taken from the Dutch results reported in the same paper.	*Cryptosporidium* spp., *Giardia* spp.	Estimated infection risk/swimming event/person in freshwater lakes: Cryptosporidiosis: 1 × 10^-4^ – 5 × 10^-4^ Giardiasis: 4 × 10^-6^ – 1 × 10^-4^	Schets [45]
Measured concentrations of *Vibrio parahaemolyticus* (qPCR) at two beaches in Southern California	Based on figures from [45, 46] for swimmers and surfers.	*Vibrio parahaemolyticus*	Average illness risk per recreation event varied by location and activity, with surfers having an average risk of illness of 3.15 × 10^-5^ and 8.2 × 10^-5^ at the two beaches	Dickinson [47]
What is the impact of storm water on the risk of recreational water related GI illness?				
Surfing in recreational water impacted by storms, based on routine faecal indicator monitoring data. Southern California marine beaches.	Surfing ingestion data [46] with a lognormal distribution with a mean and standard deviation of 3.54 and 1.80 ml/day. Based on review of the literature, volume of ingestion for swimmers was based on a triangular distribution with a minimum, mode, and maximum of 20, 35 and 50 ml/h, respectively.	Enterococci (exponential dose-response model) Faecal coliforms (Beta-Poisson model)	Based on the enterococci data, average illness rates were between 1 to 11/1,000 surfers in dry conditions and between 2 to 34/1,000 in post storm conditions.	Tseng [48]
Reference pathogens measured, by a variety of methods, in stormwater discharges.	Exposure was based on a review of the literature. Minimum, mode, and maximum ingestion was 10, 50, and 100 ml, respectively. These values were increased by 50 % for children and decreased by 80 % (irrespective of age) for low contact activities. The minimum, mode, and maximum values for duration were 0.25, 0.5, and 2 hours, respectively.	Non-typhi *Salmonella* *Cryptosporidium parvum* *Giardia lamblia* Adenovirus 4 Echovirus 12 Norovirus (Norwalk virus) Rotavirus	Norovirus was the most dominant predicted health risk	McBride [49]

The QMRA studies support the findings from the epidemiology in relation to the different results seen between point source-polluted and non-point source-polluted recreational water, as they show that risk depends on the source of faecal pollution and that, as viruses seem to be the pathogens of concern, human sewage inputs carry the greatest risk of infection. In addition, infection risks seem to be greater after rainfall.

## Conclusions

Epidemiological papers published after 2009, using both prospective beach survey and randomised control trial protocols, suggest an elevated health risk due to bathing water exposure. However, for the most part, they generally failed to quantify a distinct and credible linear relationship of the type used by regulators in the design of existing standards (an exception being the study by Wade [23] and gastrointestinal illness and enterococci measured by PCR). It would be too simplistic to attribute this failure to misclassification bias caused by the use of aggregate measures of exposure. It could be that the considerable expenditures devoted to collection and treatment of human sewage in the past 50 years in all developed nations has resulted in reductions of the principal faecal loading from the human population, which drives the pathogenic virus flux implicated in many outbreaks, symptom elevations reported world-wide, and as suggested by QMRAs. It should be noted, however, there is still a consistent elevation in illness among the bather cohort in most studies which could relate to residual microbial loadings from intermittent discharges from a sewerage system after rainfall events, polluted urban flows with impaired water quality due to improper cross-connections to the sewerage system, poorly treated sewage caused by plant overloading after population growth or sewerage extension, and, finally, human pathogens derived from livestock farming areas, generally comprising protozoan or bacterial pathogens.

Source attribution, through quantified microbial source apportionment, linked with appropriate use of microbial source tracking methods should, therefore, be employed as an integral part of future epidemiological surveys. In addition, protocols defining exposure (i.e., aggregate measures vs spot determinations) should be integrated with the design of bathing water compliance requirements. Here, many authorities are effectively moving towards aggregate measures such as percentile or geometric mean values and away from allowed thresholds. It would be wise for the research and regulatory communities to examine the impact of natural variability in environmental faecal indicator concentrations on the precision and, hence, credibility of either approach as a predictor of health risk, whether culture or molecular methods of enumeration are deployed. Finally, on the choice between culture and molecular methods, there is a clear need for re-examination of the precision and reproducibility of both approaches and their differential attenuation through common sewage treatment and disinfection systems. This is required to allow the regulatory community to make objective choices between available systems. 
